# Serotonin is a multifaceted player in the immune response

**DOI:** 10.52586/4939

**Published:** 2021-08-30

**Authors:** Ling Lin, Kebin Hu

**Affiliations:** 1 Division of Nephrology, Department of Medicine, Penn State University College of Medicine, Hershey, PA 17033, USA,; 2 Department of Cellular and Molecular Physiology, Penn State University College of Medicine, Hershey, PA 17033, USA

Serotonin (5-hydroxytryptamine, 5-HT) was initially isolated from extracts of gut enterochromaffin (EC) cells causing smooth muscle cell contraction. It was named enteramine by the Erspamer group in 1937 [1]. In the central nervous system, serotonin is produced by neurons within the raphe nuclei of brainstem, regulating various brain functions such as mood, perception, memory and stress responses [2]. Peripheral serotonin accounts for about 95% of total serotonin, is synthesized by EC cells and is subsequently released into the circulation. Platelets take it up via membrane serotonin reuptake transporter (SERT) and store it in their dense granules [2–4]. Synthesis of 5-HT is limited by two enzymatic steps: (1) generation of 5-hydroxytryptophan by tryptophan hydroxylase (TPH); and (2) conversion to 5-HT by aromatic l-amino acid decarboxylase. Degradation of 5-HT into 5-hydroxyindoleacetic acid (5-HIAA), the urine excretable form, is mediated by monoamine oxidase (MAO) [2, 3].

Serotonin executes its functions through one of 15 receptors derived from 7 receptor families (5-HTR1–7). These 5-HTRs, except the 5-HTR3 nonselective cation channel, are G-protein receptors and are involved in gua-nine nucleotide-binding protein (GTPase)-mediated signal pathways [5]. Many immune cells, including monocytes/macrophages, dendritic cells (DCs), neutrophils, mast cells, eosinophils, T cells and B cells express various different 5-HTRs [6]. Thus, peripheral serotonin plays an important role in modulating both innate and adaptive immune responses and inflammation. Most recently, serum serotonin levels have been considered a good predictor for the outcome of SARS-CoV-2 infection [7] and its potential role as a therapeutic target for COVID-19 treatment is being explored [8].

In a recent issue of this journal, Dr. Schoenichen and colleagues provided a comprehensive review of the role of peripheral serotonin in the immune response and in particular the role of platelets in its regulation [9]. They discuss the functions of serotonin in the innate and adaptive immune systems, as well as in non-immune endothelial (ECs) and smooth muscle cells (SMCs). Monocytes/macrophages, DCs, neutrophils, eosinophils, and basophils/mast cells are essential components of innate immunity. Serotonin clearly modulates multiple functions of monocytes/macrophages and promotes their production and release of chemokines. However, its effects on migration remain largely unknown. Immature and mature DCs express different 5-HTRs. Serotonin has been found to promote migration of both immature and mature DCs through different receptor-mediated pathways. The authors also discuss strong evidence that serotonin promotes neutrophil recruitment, and eosinophil and mast cell transmigration in a receptor-mediated manner. For the role of serotonin in adaptive immunity, the authors describe the complex effects of serotonin on the proliferation, survival, and migration of T cells, B cells and NK cells. However, the specific mechanisms remain to be elucidated. Non-immune cells, such as ECs and SMCs, also express various 5-HTRs and respond to serotonin to trigger contraction or modulate immune adhesion. Notably, the authors also briefly discuss the connection between the microbiome and serotonin synthesis, which is a current topic of great interest.

In summary, there is strong evidence supporting the multifaceted roles of serotonin in regulation of the immune response. However, underlying mechanisms remain to be elucidated, and more mechanistic studies are warranted in order to further our understanding of the complex effects of serotonin on immune responses and inflammation.
